# Lessons on maintaining assessment integrity during COVID-19

**DOI:** 10.1007/s40979-022-00112-1

**Published:** 2022-07-28

**Authors:** Samar Yakoob Almossa, Sahar Matar Alzahrani

**Affiliations:** English Language Centre, Umm Al-Qura University, Makkah, Saudi Arabia

**Keywords:** COVID-19, Online learning, Emergency online teaching, Higher education, Online assessment

## Abstract

In an era where conditions for education are rapidly changing globally, online assessment presents several opportunities as well as challenges in the higher education landscape. The forceful transition from face-to-face to online assessments, as part of the emergency implementation of online learning due to the COVID-19 pandemic, has affected teaching, learning, and assessment experiences worldwide. This study explores how faculty members in Saudi universities secured their online assessment during phase one of the COVID-19 pandemic. The research aims were: 1) identifying faculty assessment practices before the onset of COVID-19 and comparing these with practices during the pandemic, and 2) identifying the major challenges faced by the faculty members of the study in securing their online assessment to ensure that academic integrity and assessment standards remained intact. Data were collected from seven university professors through a self-reporting survey, followed by semi-structured interviews. The faculty members found the pandemic period to be the best time to change their assessment methods, and incorporate innovative ideas that conformed to both their own beliefs and students’ needs. The factors that influenced the faculty’s assessment alterations were their personal beliefs and learnings from others’ experiences, in addition to the guidelines issued by Saudi Arabia’s Ministry of Education. The results of this study have implications for the development of post-COVID-19 assessment practices and professional development priorities.

**Supplementary Information:**

The online version contains supplementary material available at 10.1007/s40979-022-00112-1.

## Description of the study

This study reports faculty members from Saudi higher education context experience with securing assessment integerty during COVID-19. It sheds the light on how individual and collectives experiences provide data on the possible means for securing assessment practices during such a sudden transition.

## Introduction

Faculty assessment practices are an essential part of the teaching experience in the context of higher education (HE). They have an impact on learning and teaching, faculty knowledge, understanding, and practice topics of inquiry. As such, they are vital in light of the decisions based on students’ assesssments results made for futuer education and employement opprounities and their consequent impact on students’ future choices. The policies of the Ministry of Education (MoE) influence assessment practices in the Saudi HE context, as they state that 60% of assessment is to be based on examinations and 40% on final examinations or other types of assessment, such as mid-term examinations or quizzes (Saudi Universities bylaws 1996). Assessment policy in Saudi HE places great emphasis on examinations—which is clear in the accreditation documents (NCAAA 2015:15)—and assessment is generically referred to as ‘examinations and other assessment tasks’, but without clear specification of these other tasks. During phase one of the COVID-19 lockdown, which coincided with the first week of mid-term exams in Saudi universities, the policies of the MoE shifted. It released several statements and memos, encouraging the adoption of alternative assessment methods and allowing a change in the weight accorded to mid-term and final examinations, and assessment tasks (AlRiyadh Newspaper 2020). The MoE issued a memo in April 2020 that outlined the temporary changes, and suggested that final assessment or examination account for only 20% of a grade, and alternative assessment methods be sought. Some universities banned final examinations in the written format to encourage the use of alternative assessments and avoid technical difficulties reported by students, making use of the assigned distribution of 80% for coursework and 20% for final assessment or examinations (AlRiyadh Newspaper 2020).

The transition to online learning and teaching in Saudi universities occurred within a few days, as all had the necessary technical infrastructure (e.g., Blackboard and other apps) for teaching and communication. Faculty members and students in Saudi HE continued the semester with several changes (Almossa 2021; Almossa and Alzahrani 2022). The challenges reported involved the lack of certainty surrounding how long the situation would last, how assessment would be shifted, how deadlines and submissions would be amended, and how examinations would be postponed or substituted with alternative assessment tasks. Other issues were related to internet infrastructure and access to equipment’s and tools. In a study on university students’ engagement with learning and assessment during COVID-19 in Saudi HE, and the impact of sudden changes in assessment policies on their experiences, the main challenges reported by the students during the phase included technical problems, communication, assessments, and personal issues such as mental health (Almossa 2021). In Almossa (2021) study from the Saudi context with student participants, it was reported that examination issues significantly impacted students’ assessment experiences. Questions have been raised—as the pandemic is ongoing at the time of writing this paper—on fair methods of student assessment, the COVID-19 pandemic’s lasting impact on assessment, and the sustainability of pandemic-related changes in the post-pandemic period; calls have been made for reflections on new, innovative online assessment methods that can suit different courses (García-Peñalvo et al. 2020; Al-Salman and Haider 2021). Another research project linked to this study focused on faculty assessment practices in Saudi universities concluded that the participants (Almossa and Alzahrani 2022) has similar ways of thinking and endorsing assessment during the pandemic (Almossa and Alzahrani 2022). Yet there is more to learn about how faculty members design and secure their assessments, and to what extent integrity challenges influence their assessment practices. Accordingly, this study examines HE faculty practices in relation to ensuring academic integrity when conducting assessments during the pre-COVID-19 period, and the pandemic-induced transition to online assessment. We document the experiences of seven faculty members from a sociocultural perspective that sheds light on individual experiences across colleges, subject areas, expertise, and contexts.

## Literature review

### Assessment practices during the COVID-19 pandemic

In 2020, the COVID-19 pandemic necessitated the implementation of emergency remote teaching and learning—an immense educational experiment—as governments worldwide prioritised the health and safety of teachers and students (Berry et al. 2020; Kamenetz 2020). It marked the beginning of a critical era with respect to implementation of education reforms and efforts to make emergency changes sustainable, as the pandemic rapidly affected the provision and reception of education worldwide. The shift of university teaching/learning and assessment to an online mode was an obvious outcome, and has come to be identified and referred to as ‘emergency e-learning’ (Murphy 2020:492) or ‘emergency remote teaching’ (Bozkurt and Sharma 2020, i). The assessment of students’ performances in unexpected and unusual circumstances is a challenge, in this case associated with the urgent need to modify assessment methods to transition successfully to online education. The transition to online learning occurred within a matter of days in March 2020, and significantly affected the long-term future of online learning and assessment. Emergency e-learning was the obvious option to provide access to synchronised and asynchronised education, and helped maintain the learning environment during the pandemic. At the time that this paper was written, online learning (e-learning) had become the new normal, rather than an emergency measure, and could thus be considered the second phase of e-learning implementation (Murphy 2020). However, it is unclear how long this new method of learning and teaching will last.

As the shift to e-learning was forced and unplanned, bringing about radical changes, several questions have been raised on how HE faculty members handled online assessments during the sudden transition to online learning. For instance, how did they maintain some or all assessment standards (e.g. assessment purposes, design, fairness, and measurement theory) in comparison to their pre-COVID-19 assessment practices? Pauli et al. (2020) reported on the future of assessment, focusing on five principles and targets for 2025. The five principles that they shared regarding technology-transformed assessment are to ensure that an assessment is authentic, accessible, appropriately automated, continuous, and secure. The recent changes in assessment have required staff to develop digital skills and, thereby, enhance their ability to experiment with and implement innovative online assessment practices. Recent studies report the presence of challenges in adapting to a new teaching/learning/assessment mode, such as technological hinderances and incompetence of faculty and students (García-Peñalvo et al. 2020; Guangul et al. 2020; Watermeyer et al. 2021). In large studies conducted in UK universities, Watermeyer et al. (2021) surveyed 1,148 teaching staff members across all majors and career stages. They reported significant dysfunctionality and disturbances in teaching, overwhelming experiences in dealing with technological tools, and trouble with the rapid transition to digital pedagogy. Additionally, there was a reported increase in students’ needs and demands to contact their instructors. However, this article did not highlight their assessment practices. Although online teaching itself has many challenges, an online assessment has often been considered as the biggest obstacle to completing the academic year (García-Peñalvo et al. 2021). As Huber and Helm (2020) suggest, pre-existing knowledge of different situations and contexts should be linked to the current situation. Therefore, it is necessary to collect and assess information on teaching, learning, and assessment during the pandemic from across various contexts, situations, and educational levels using qualitative and quantitative research methods.

### Assessment integrity during the COVID-19 pandemic

Assessment integrity is an important part of ensuring a sound assessment process and accurate results, while assessment design is an important part of securing assessment to prevent instances of cheating and plagiarism. Some types of assessment tasks minimise the chance of academic misconduct, such as those that focus on application of learning rather than reproduction of knowledge (Ellis et al. 2020; Brown and Janssen 2017).

Securing online assessment against academic misconduct was a major concern during the pandemic. Eaton (2020) noted that the pandemic is a time wherein technology companies with advanced proctoring and cheating measurement systems thrive with solutions for institutions, but these require infrastructure such as laptops equipped with cameras and internet access, programmes for students and faculty, and substantial budgets. Several studies reported the measures taken by faculty members and students in this scenario. In their study, Khan et al. (2021) remarked that faculty members transformed the challenges into a window for innovative assessment designs. Institutional support for such collective efforts and practices and sharing of ideas are important to the continuity of such innovation in assessment practices.

Some universities in Saudi Arabia limited options for the delivery of online lectures through specified platforms only, such as Blackboard and Webex to monitor teaching activities and to ensure the security of the data and the protection of the students’ data protection. Others did not use online proctoring software that required camera use or real time invigilation (Almossa 2021; Meccawy et al. 2021).

Various methods were employed to transform traditional assessment to suit online assessment. These included time-restricted assessment tasks such as quizzes, non-restricted assessments such as home assignments, essay questions, video streaming assessment tasks such as oral examinations, and viva voce presentations (Gamage et al. 2020). Creating equal opportunities for students to undergo online assessment was not always possible, given individual needs and inequality in access to equipment and internet connectivity (Almossa 2021). Other questions were raised as to how a faculty member could tell whether a student had left the exam because of a technical issue, or lack of competence in the subject.

Dawson (2020:19) noted that assessment security refers to ‘*measures taken to harden assessment against attempts to cheat. This includes approaches to detect and evidence attempts to cheat, as well as measures to make cheating more difficult’.* Academic integrity encourages students to work on an assigned task and value what they do, creating an environment wherein policies, procedures, and pedagogies that discourage cheating are upheld (Dawson 2020). Maintaining assessment security is a process that involves being secure about one’s judgements and the consequences thereof, meaning that students will perform assessment tasks authentically and the outcomes will be a true reflection of their performance. Hence, assessment security requires controlling the conditions under which assessment tasks are performed to meet these criteria.

### Measurement of assessment practices

Several measurements have been developed to examine assessment practices. These vary from classroom observations, self-reporting instruments, and interviews, to surveys (for a full review, see Gotch and French 2014). DeLuca et al. (2016) introduced the Approaches to Classroom Assessment Inventory (ACAI), which incorporates contemporary assessment standards. The ACAI enables educators to determine their assessment practices and serves as a useful tool for educational researchers to examine various assessment practices and teachers’ assessment literacy. The ACAI’s four-dimensional framework for assessment literacy is based on an analysis of 15 contemporary assessment standards (from 1990 to the present) from six geographic regions (the United States, Canada, the United Kingdom, Europe, Australia, and New Zealand). The four dimensions encompass the following aspects: (1) assessment purposes, which include assessment for, of, and as learning; (2) assessment processes, which include design, scoring, and communication; (3) assessment fairness, which focuses on standardisation, equitability, and differentiation; and (4) measurement theory, which focuses on reliability, validity, and a mixture of both (DeLuca et al. 2016). The current study uses ACAI (DeLuca et al. 2016) as an analytical framework to explore faculty assessment practices and measures taken to secure assessment during the COVID-19 pandemic.

Although several studies discussed the impact of COVID-19 on teaching in general, and on learning experiences and assessments specifically, limited research provides detailed accounts of faculty members’ experiences with online assessments during the emergency transition to online teaching/learning in HE (e.g., Meccawy et al. 2021; Khalil 2020). The changes caused by the pandemic have significantly altered educators’ assessment practices and professional development requirements. Hence, research on how these changes in assessment have affected educators is critical to understanding how to support online HE educators. Given the gaps already mentioned, this study adopts a qualitative approach to investigating faculty assessment practices during the COVID-19 pandemic and examines the impact of the sudden transition to the online mode on assessment experiences.

## Methodology

### Research questions

This study addresses the following two research questions:

1. Which practices were employed by teaching staff during the COVID-19 pandemic to secure assessment?
2. What were the main challenges faced by the participants of the study in securing their online assessment, to ensure that academic integrity was maintained during the pandemic?

#### Research context

An in-depth qualitative inquiry was conducted with seven teachers at different stages of their careers (from teaching assistant to associate professor) in the HE context, to elicit a detailed account of their approaches to maintain assessment integrity and security during the pandemic. The participants were invited to reflect on their assessment practices, with questions on the decisions they made and the effect of the transition to the online mode on their assessment practices.

#### Participants

Ethical clearance was obtained from the authors’ university under grant number 19-EDU-1–02-0005, as well as from the invited universities to adhere to their rules and procedures. Approval to distribute the survey through Saudi universites deanships of research was obtained as well.

In the first phase of this study, the last question in the provided questionnaire invited participants to tick a box if they would like to be interviewed afterward (full questionnaire results were published in the following paper: Almossa and Alzahrani (2022), with a box to leave contact information included. Those who ticked that box were then shortlisted for the interviews. Only those who met the criteria (including department type, age group, and field of study) were contacted.

Seven (three male and four female) faculty members, working in the HE sector in Saudi Arabia, participated in this study; they were informed that participation was voluntary and they had the right to withdraw at any time. All of them had taken a course in assessment during their teaching career, and used various assessment methods before and during COVID-19 pandemic. Table 1 summarises the participants’ demographic data. Their years of experience ranged from 6 to 11, and they taught Bachelor’s, Master’s, and PhD students. The teaching positions included those of teaching assistant (1), lecturer (1), assistant professor (4), and associate professor (1). Their disciplines consisted of business management, theology, demographic studies, mathematics, information science, and educational planning management.

**Table 1:** Participants’ demographic data

	AHMED	NOOR	SAEED	MONA	AMEERA	NASSER	RAID
Position	Lecturer	Teaching assistant	Assistant professor	Assistant professor	Assistant professor	Assistant professor	Associate professor
College	Business College	Theology College	Social Sciences College	Islamic Studies College	First Common Year Deanship	Computer Science College	Education College
Disciplines taught	Business management	Shari’a	Demographic studies	Islamic studies	Mathematics	Information science	Educational planning and management
Career stage	6–10 years	3–5 years	Over 11 years	Over 11 years	Over 11 years	6–10 years	6–10 years
Degrees taught	Bachelor’s	Bachelor’s	Bachelor’s; Master’s; PhD	Bachelor’s; Master’s	Bachelor’s	Bachelor’s	Master’s; PhD
Main assessment practices used in the previous semester (1441/2020) before the pandemic	Assignments; projects, quizzes; mid-term and final exams; presentations	Formative assessment; summative assessment; assignments; quizzes; mid-term and final exams; presentations	Formative assessment; summative assessment	Summative assessment; assignments; quizzes; mid-term and final exams; presentations	Assignments; quizzes; mid-term and final exams	Assignments; projects	Assignments; projects; mid-term and final exams; presentations
Changes in assessment methods made during COVID-19	Assignments; quizzes; presentations; portfolio	Open-book exam	Assignments; quizzes; mid-term and final exams; presentations	Assignments; open-book exam; oral discussion	Assignments	Assignments; project	No change in assessment practices during COVID-19

Participant names have been anonymised

### Instruments

The questionnaire was divided into four parts: participants’ demographic data (see Table 1), assessment methods followed before and during the COVID-19 pandemic, assessment practices, and professional development priorities (the participants’ answers were followed-up on during interviews). The semi-structured interview guide focused on the study’s main themes as follows: part one – assessment during COVID-19; part two – impact of the pandemic on assessment practices (changes and alterations caused in assessment plans); part three – assessment practices, purposes, processes, fairness, and measurement theory.

### Data collection

This study was conducted in two phases. In phase one, all faculty members of Saudi universities received a questionnaire inviting them to participate in the study (a discussion on the full survey results is outside the scope of this paper, which only addresses the seven interview participants’ responses). Subsequently, the seven participants were selected for interviews using purposeful sampling according to the following criteria: willingness to participate in interviews; availability during the interview period; appropriateness of survey answers; and representation of different fields, career stages and positions, and gender identity. They were invited for individual interviews to further discuss their answers to the survey questions and obtain more information on their individual experiences with assessment during the pandemic, along with the latter’s impact on their practices. The participants were informed that the interviews were recorded solely for research purposes, assured of their anonymity, and told that they had the right to withdraw consent at any point. All the interviews were conducted online using Webex; by the end of the summer of 2020, the Webex meeting was scheduled and agreed on by all the participants. Each interview lasted 30–70 min. The relevant mp4 files were downloaded then transcribed verbatim in Arabic, the native language of all of the participants. They were finally translated to English during the write-up process.

#### Data analysis

All interviews were transcribed, organised, and analysed using the MAXQDA software, following Bryman’s (2016) guide on interview analysis. Thematic analysis was performed to code the main themes that emerged from the interview data. First, the second author read all the interviews several times, coded the data, and then reviewed the data against the interview guide to compare the focus of study with the topics that emerged during the interviews. The data were grouped under the appropriate codes (sample of codes provided in Additional file 1: Appendix A). The coding scheme was revised and modified several times to make sure the codes accurately reflected the data. In the next stage, the main themes that emerged were identified, and the data were grouped into categories. The first author double-coded the data to ensure inter-coder reliability.

## Results

### Which assessment practices were employed by teaching staff during the COVID-19 pandemic?

#### Assessment purposes

The participants relayed that assessment purposes during COVID-19 were focused on summation of students’ levels of learning and assigning grades. Some of the participants reported making less use of formative assessment, as the focus was on finding alternative ways to assess the students and assign grades. The data acquired as a result of surveying teaching staff in Saudi HE—which is beyond the scope of this paper—showed that formative assessment practices were almost demolished during the pandemic, given that the participants had to focus on finalising graded assessment tasks to release total coursework grades, as required by the MoE (Almossa and Alzahrani (2022).

Six out of the seven participants mentioned changing their assessment practices during the pandemic, to align with the MoE guidelines and the shift to online mode. Two out of the seven participants said that they used formative assessment before the pandemic, while the other five mentioned using mid-term and final examinations, assignments, projects, quizzes, and presentations. During the pandemic, the respondents followed the rules and guidelines specified by the MoE. The MoE had raised the percentage of coursework to 80% of the total assessment and gave the teaching staff a month to design, implement, accord scores, and share the results with students. This left three weeks for withdrawal before the final assessment, which was worth 20% of the student’s final grade. The assessment methods participants used during the COVID-19 pandemic included assignments, presentations, portfolio, quizzes, open-book examination, projects, oral discussions, and mid-term and final examinations.

#### Assessment processes

The participants reported focusing on assessment design, scoring, and communication. They wanted to create alternative assessment tasks that were reliable, but found this challenging due to the limited time available for preparing and conducting the assessment tasks, number of students, and their lack of preparedness for new assessment methods.

The participants mentioned that, in the pre-COVID-19 era, they had used various assessment methods as per their department’s regulations and compliance with the MoE (60% for the final examination, and at least 40% for the mid-term examination and coursework). These included projects, research papers, assignments, and open-book examinations. Further, they stated that their assessment practices had undergone variation due to the pandemic’s impact on their teaching, learning, and assessment experiences. All the participants mentioned that online assessment facilitated more freedom in using various assessment types. It also facilitated greater flexibility in introducing new ideas through different types of questions in examinations (including both multiple-choice and true–false questions), mark distribution (which was fixed earlier), and deciding on assessment tasks. The second participant, NOOR, who was a teaching assistant in a theology college, spoke about how the pandemic broke the department’s restrictive traditional assessment rules: ‘*We now have the freedom to use various assessment strategies, that were not even considered as options before the pandemic. We have the freedom to give research projects, group tasks, open-book exams, and more.’* Yet, the freedom to redesign assessment came with challenges and restrictions, as the participants reported.

The freedom in assessment design, implementation, and scoring was an advantage that participants appreciated, regardless of the hardships. Another advantage of online assessment was how quickly and accurately Blackboard corrected examinations and produced instant statistical post-examination reports. This was not as easy before the pandemic, when exams were paper-based. Clearly, the use of technology enhanced the process of administration, correction, and reporting, by enabling the instant release of grades and feedback. NASSER, an assistant professor in a computer sciences college, explained the convenience of correcting students’ assignments on his phone: ‘*I downloaded a Blackboard instructor app on my phone, which made it easier to monitor my students’ submissions. When I receive notifications of submissions, I correct the assignment on my phone.’*

Two out of the seven participants reported no change in their assessment practices because they taught graduate students, and used the same assessment methods that they had followed before the pandemic. RAID, an associate professor in an education college, expressed this view, as she did not experience fundamental changes in her practices. Instead, she only experienced a lack of face-to-face interaction to assess students:

*‘As a postgraduate professor, I do not feel any fundamental changes have occurred. Except for the fact that I could not see my students face-to-face and changed some presentation assessment criteria, I did not experience any other changes. I gave them an open-book exam and 24 hours to finish it to assess their high-order skills. I assessed how they analysed the information, linked them, gave their points of view, and showed their creativity.’*

#### Assessment-related communication

All the participants reported that online learning offered more channels for online assessment-related communication with their students, such as discussions on the requirements for and expectations from assessments. While some students resisted changes in assessment methods or types and many felt overloaded by assessment demands, several staff members remarked on the positive changes in their students’ behaviour; for example, they noticed that some students had become more confident and better communicators (more active in discussions and small talk), showed greater commitment to their studies, and improved their performance in assessments after attending online classes. Further, some of the participants mentioned that their students’ understanding of the subject and performance in assessments had improved, thereby improving the overall course learning outcomes. However, five participants stated that they missed maintaining personal contact with students during examinations; for example, being in the lecture or examination hall to interact with students and invigilate examinations, seeing their facial expressions, and having small discussions with them. Students were not required to keep their cameras open during lectures or examinations, which also increased the likelihood of cheating.

The students found themselves in a situation wherein they had to learn how to work on assessment tasks they had not been previously introduced to or trained in, such as writing research papers. AHMED—a lecturer in a business college—noted the following transition from traditional to alternative assessments, and how difficult it was to introduce and communicate the new tasks to students: ‘*They found the methods vague, and had lots of questions. If I ask them to do a simple research project, not a real one, I just ask them to collect information about a topic. Students ask for written guidelines (step-by-step documents) to do the tasks. They were scared of this new experience at first, but after discussions and completion of the tasks, they gained confidence.’*

The participants also noted that communication from their departments could be improved for better conveyance and clarification of information on the expectations of their students and for the freedom to act upon them without restrictions. MONA believed that faculty could have benefited from a clearer departmental plan.

#### Assessment fairness

The participants shared their views on fairness and concerns of academic integrity. NASSER mentioned that cheating was easy—not only in examinations, but also in assignments and projects—so he approached it by discussing the results with his students in order to determine who had actually understood the subject, and who had cheated. This process, as he described it, had a negative side: ‘*Students who gained marks that they didn’t deserve would learn a lesson from the open discussion in front of their classmates, but this is exhausting for us [faculty].’* In response to the question on how he achieved fairness in examinations, the answer was*, ‘I choose one day to conduct exams for all the groups, to achieve fairness, so they don’t copy the questions. But with e-learning, they can capture the screen. Even though the questions are randomised, parts of them can be revealed.’* This meant that, regardless of having a randomised question bank, some questions would be similar and possibly leaked between students.

NOOR mentioned several practices that were followed to ensure that assessment practices were standardised, differentiated, and equal. She articulated her approach as follows:

‘*I differentiate between assessment methods. First, I don’t put the biggest weight on examinations, as some students are not good at taking exams. Second, I like to ask my colleagues about possible assessment strategies, and test these before implementation. If I notice any issues in students’ performances, I make changes with the second group. Third, students’ results give me an indication of whether the assessment tool is fair. If it is not fair, I assign other methods for students to get grades. I also use their feedback to improve the exam questions.’*

NASSER and NOOR stressed the role of collaboration with colleagues and students in designing and delivering fair assessments, and evaluating assessment tools for future development. The awareness and commitment that they showed prove that the exercise of care in their practices had developed with accumulated teaching experiences and constant consultation. Meanwhile, SAEED relied on assignments as he found them the best way to achieve fairness during online assessment, and MONA believed that following clear assessment standards were an important part of ensuring fairness.

#### Measurement theory

The participants listed the use of several methods to achieve reliability and validity in their assessments, such as reviewing questions and collaborating with colleagues. NOOR described her approach:

*‘I use specifications to make sure I write balanced questions, in terms of numbers and percentages from all the lectures. I compare my students’ coursework grades with their final exam grade. I also compare their grades with those of my colleagues’ students, because it gives me an indication of their level knowledge and any possible problems. Also, I give my colleague a random sample to correct, and ask her for a report.’*

In the same vein, SAEED described his process of writing multiple choice questions—reviewing the questions and answer options—to ensure that the questions and options were clear, fair, and suitable. Both he and NOOR exchanged examination samples with their colleagues to check and review them for suitability.

### What were the main challenges faced by the participants in securing their online assessment to ensure academic integrity was maintained during the pandemic?

The participants were asked about the challenges they faced in redesigning and administering assessment tasks during the pandemic to be able to secure these tasks. The participants reported an increase in the workload, as a result of the efforts to secure assessment. In this process, there were several concerns of academic integrity, assessment security measures, technical issues, inadequate training, and vague guidelines.

#### Increased workload to secure assessment

The increase in academic workload, due to the demand of securing assessment, was a noticeable factor that affected the participants’ experiences. Securing the assessment process included redesigning tasks to fit the new mode and putting certain measures in place to minimise cheating. AHMED’s opinion was seconded by SAEED, an assistant professor in a social sciences college, who found that online assessments had increased the teaching staff’s workload since they had to introduce students to new assessment methods and tasks, encourage them to examine various options, attend to their questions and concerns, and work with colleagues to complete the tasks. He added that group work between students was also an extra workload, saying, ‘*We replaced the final exam with projects, and that added to our load as students could not interact physically, hindering their team building. I found that in group tasks, only one person does all the work.’* Further, participants of the study who were very interested in using innovative assessment methods also felt the increase in workload, but insisted on using accurate and varied methods despite facing several limitations in online learning and teaching during the pandemic.

#### Securing assessment

Securing online assessment by conquering online cheating was one of the foremost challenges and disadvantages of online assessment, as reported by participants who were concerned about academic integrity. An important point to note is that all the participants conducted assessment and examinations without live proctoring (i.e., neither cameras were opened nor online invigilation was implemented, thereby increasing instances of cheating). NOOR shared her views as follows:

‘*There were negative sides to online examinations. I could not judge the credibility of the marks. It took me time and effort to check if cheating had occurred in an exam. In some cases, I knew the level a student was at and hence was surprised by her mark in the exam. I could tell that some of them had cheated by comparing their answers. Comparing answers in the online mode is difficult than comparing on papers. In some cases, I had to call the student to check if cheating had happened or not*.’

When the participants were asked about how they managed to secure assessment and minimise cheating, MONA, an assistant professor in an Islamic studies college, answered that there was a need to build a huge question bank to reduce the risk of cheating attempts in addition to other measures, such as giving students different versions of the questions. NASSER mentioned that he had learned from the first phase, and thus created a question bank during the summer: ‘*I overcame negative outcomes during the summer course by making a question bank: true and false, multiple choice, essay questions, et cetera. The questions are varied, and show students’ individual differences. I created 200 questions; 20 questions are randomly chosen for each student.’*

One of the challenges was the issue of students creating groups for cheating. Some students made use of not just the available access to resources, but also to each other for answering exam questions. NASSER noted that one of the reasons behind easy cheating was the lack of or poor training of the teaching staff, so they chose the easiest options in the absence of guidelines to inform their decisions. Online learning and assessment are powerful tools, but the proper use of this technology is yet to be learnt. Therefore, participants stressed the importance of policymakers issuing adequate training and guidelines. In fact, the major limitation mentioned by the participants was the gap in online learning due to lack of clear guidelines and sufficient supporting documents*.* NASSER shared this view as follows: ‘*They tell us to have mercy on students; we also want to measure their learning and improve it. But the skills and technical support are poor. I suggest they add a Q and A section, and advanced-level documents.’*

#### Technical issues

While some of the teaching staff considered correction and scoring in online assessment correction a convenience, the use of phones to take online examinations was reported as being troublesome by many students who did not have other options, such as a laptop or tablet. SAEED mentioned that some of the technical issues faced by his class were caused by his students’ complete reliance on using their phones. According to SAEED, ‘*Students were supposed to have a laptop and speedy Internet connection, but, unfortunately, they pay 4000 Saudi Riyals for a phone but not 1000SR for a laptop. We faced problems during exams because they used their phones, even though the guidelines said they had to use a computer.’*

AMEERA also mentioned that the differences in internet infrastructure among students made the entire online teaching/learning process difficult. To overcome this issue of unfairness, due to privileges or lack thereof, she suggested facilitating online assessments on campus in the future, stating that, *‘exams can be conducted online but in the university labs, where an excellent network is in place and there are technicians to support and solve problems instantly.’*

## Discussion and conclusion

This study focused on HE university teaching staff’s experiences with securing online assessment during the pandemic in the Saudi context. By focusing on staff perspectives, we were able to examine how sudden changes in assessment affected their students and assessment practices.

The sudden COVID-19-induced transition to online learning, teaching, and assessment presented the participants with new challenges in designing, implementing, and correcting assessments with the use of technology that some of them had never used before in full-time teaching. This finding is consistent with narratives found in current literature, that educators and students faced the transition with both positive and negative emotions (Eringfeld 2021; Naylor and Nyanjom 2021). The data revealed changes in assessment practices that were guided by the MoE policies, guidelines, and departmental implementation plans. Participants reported both positive and negative experiences during the early phases of the transition to online assessment, as several studies had earlier indicated as well (Guangul et al. 2020; Watermeyer et al. 2021).

The challenges in conducting online assessments included technical issues, departmental restrictions (e.g., certain assessment types and questions), reduced response to training needs, issues in securing online assessments, and the lack of assessment creditability. These findings are in line with Guangul et al.’s (2020) report that online infrastructure and academic dishonesty presented teaching faculty with various challenges. Preventing cheating was identified as one of the major challenges in online assessment, as reported in previous studies (Xu and Mahenthiran 2016). Cheating and plagiarism in online assessments are easy, effortless, and frequent (Gathuri et al. 2014; Hillier 2014; Mellar et al. 2018). Participants relied on producing sizable question banks with several versions when it came to exams, and using other objective assessments while there was lack of infrastructure in securing online examinations, such as online proctoring. Measures to prevent and minimise cheating are costly and require infrastructure, for which the institutions were not prepared. There were no cameras or invigilation for online examinations, which made cheating more accessible. In a recent study on the impact of COVID-19 on university students, Almossa (2021) reported that students found different ways to cheat during exams, such as using WhatsApp groups or relying on those who took the exam first to give them the answers. Although challenges to academic integrity were obvious and exams without proctoring did not work, the teaching staff still administered online exams. This raises the question: why conduct exams when they are not secure or protected, given that unsupervised tests facilitate cheating and cause a false increase in level of performance (Dawson 2020)? Participants cited reasons for not shifting to alternative assessment methods including existing limitations—such as lack of clear guidance, large number of students in the classroom thereby making other methods of assessment impractical—and limited time available for designing and implementing alternative assessments. Using in-person pre-COVID assessment designs was the only option for some faculty members, which has been reported in previous studies (Dietrich et al. 2020; Eaton 2020; Rupnow et al. 2020). In contrast, Guangul et al. (2020) reported from the Omani context that most of the respondents and college faculty preferred un-proctored assignments and project-based assessments for practical reasons, such as preventing cheating and overcoming lack of appropriate infrastructure for online proctoring. García-Peñalvo et al. (2021) suggested using various continuous assessment tasks, such as presentations, papers, exercises, and videos, while reserving online proctoring for the final exams, when there is no alternative for a large group assessment. However, this requires staff training and investment in software. Even with new assessment tasks, participants reported that introducing students to these was a time-consuming process. This is echoed in Khan et al.’s (2021) findings.

In the same vein, Meccawy et al. (2021) suggested not only using measures and precautions to prevent cheating, but also providing the faculty with extensive training on cheating methods and techniques. In addition, they stressed the role of stakeholders in establishing strict punishments to deter cheating. Another suggestion was conducting online assessments on university premises. This was also one of the solutions suggested by one of our seven participants.

These findings show that examination security and incorporation of manageable assessment tasks are important topics to consider in the development of teaching staff’s online assessment literacy. The participating teaching staff reported that, while they had attended some workshops, implementation was not easy and required tailored training. They needed departmental support in considering the capacity of online classes, which in turn can affect assessment and its security. In the same vein, García-Peñalvo et al. (2020) suggested introduction, training, and support of teaching staff and students to work on different assessment tasks, in addition to changes in departmental policy. The importance of training was also echoed in Sasere and Makhasane’s (2020) study recommendations, in which they investigated HE virtual teaching delivery and assessment during the COVID-19 pandemic.

Limitations of the study include that in this study we used self report throught data which provides more subjective data and future research could look into the actual documented effects of assessments by using available data on assessments during the COVID-19 lockdown from universities compared with the data from pre-COVID-19 assessments.

The current study suggests several implications: 1) implementing changes in course specifications for better adaptation to the new normal, that is, online education (i.e. all departments should update their course specifications and develop assessment practices); 2) ensuring that students do not cheat by conducting examinations in the university labs (students take the examination online, but can be invigilated to maintain the efficacy that online correction offers); and 3) providing tailored training guidance to address the needs of teaching staff (for instance, training on how to use technology in distance education).

## Supplementary Information

Additional file 1: Appendix A.

## Abbreviations

HE: Higher Education
MoE: Ministry of Education
ACAI: Approaches to Classroom Assessment Inventory
