# Supplementary material for: Lessons on maintaining assessment integrity during COVID-19
Source: Int J Educ Integr. 2022 Jul 28;18(1):19. doi: 10.1007/s40979-022-00112-1 (PMC9330967; doi:10.1007/s40979-022-00112-1)
Supplement: Supplementary file 1 — Additional file 1: Appendix A. [file 40979_2022_112_MOESM1_ESM.docx]

**Appendix A**

Interviews Codes Samples

|  | **Codes for the assessment practices BEFORE COVID-19** | **Number of times mentioned in the data** |
| --- | --- | --- |
| 1 | Various types of assessment | 3 |
| 2 | More and stronger communication | 1 |
| 3 | Better and more assessment methods | 3 |
| 4 | Open book final exam | 1 |

|  | **Codes for the assessment practices DURING COVID-19** | **Number of times mentioned in the data** |
| --- | --- | --- |
|  | **(Positive)** |  |
| 1 | More communication channels | 1 |
| 2 | Better assessment outcomes | 2 |
| 3 | More experienced teachers on technology use | 3 |
| 4 | Quick electronics assessment work | 3 |
| 5 | More flexibility in mark distribution | 1 |
| 6 | Paper saving | 1 |
| 7 | More teacher satisfaction of students’ understanding | 1 |
| 8 | Blinded review | 1 |
| 9 | Easy access to information | 1 |
| 10 | No big change in assessment | 4 |
| 11 | More open discussions for feedback | 1 |
| 12 | More students’ commitment | 1 |
| 13 | Various types of questions | 3 |
| 14 | Various types of assessment | 5 |
| 15 | More confidence | 1 |

|  | **Codes for the assessment practices DURING COVID-19** | **Number of times mentioned in the data** |
| --- | --- | --- |
|  | **(Negative)** |  |
| 1 | Missing the positive impact of personal contact with students | 5 |
| 2 | Less students’ participation and interaction | 3 |
| 3 | Technical issues with online exams | 3 |
| 4 | More load on teachers | 2 |
| 5 | Blinded review | 1 |
| 6 | One type of questions in exams | 2 |
| 7 | Lack of credibility | 1 |
| 8 | Less assignment and tasks | 1 |
| 9 | Students resist new types of assessment | 2 |

|  | **Codes for challenges** | **Number of times mentioned in the data** |
| --- | --- | --- |
| 1 | Does not support drawing on maps | 1 |
| 2 | Students miss the practice of writing | 1 |
| 3 | Lack of score credibility and more cheating | 7 |
| 4 | Teachers need training and guidance | 2 |
| 5 | Supports only one type of questions in exams | 2 |
| 6 | Technical issues | 1 |
